# Mapping a Course for PFCs: Transfer Between Mothers’ Milk and Serum

**Published:** 2007-02

**Authors:** Scott Fields

Studies have found assorted perfluorinated compounds (PFCs)—the persistent chemicals in such products as nonstick coatings—in samples of human blood and milk, but what isn’t clear is how efficiently the chemicals transfer between these two media. To address this gap, researchers in Sweden compared PFC levels in blood serum and milk samples to better understand the lactational transfer of these compounds **[*EHP* 115:226–230; Kärrman et al.]**.

Previous animal and human studies have shown that mothers can pass certain PFCs to fetuses and infants. That these compounds can find their ways into humans at the earliest stages is cause for concern because the PFCs perfluorooctane sulfonate (PFOS) and perfluorooctanoic acid (PFOA), which have infiltrated ecosystems from Asia to Antarctica, have been linked in laboratory animals to effects that include liver and testicular cancer, developmental defects, immune disruption, neuroendocrine effects, and birth defects.

The team collected milk and blood samples from 12 women at three weeks postpartum. The team also compared PFC levels from this relatively small sample to levels in human milk samples collected from 1996 through 2004 from groups of 25 to 90 women per year.

The team found eight PFCs in the current serum samples and five in the current milk samples. All of these milk samples contained PFOS (which was also the compound with the highest mean concentration) and perfluorohexanesulfonate. Some also contained PFOA, perfluorooctanesulfonamide, or perfluorononanoic acid. These patterns and levels were similar to those detected in the earlier milk samples.

The scientists calculated that the breast milk PFC concentration averaged about 1% of the corresponding maternal serum concentration. They write that the estimated levels of PFCs that infants received from mothers (about 200 ng per day) could represent a substantial exposure, and call for further studies of the potential hazards of PFCs in breast milk.

They also found that the relationship between serum and milk PFC levels depends on the specific compound. These differences, the scientists caution, may not necessarily indicate the efficiency at which the different compounds travel from whole blood to milk. Variables such as how readily each compound concentrates in blood plasma rather than whole milk may affect the ratios.

## Figures and Tables

**Figure:**
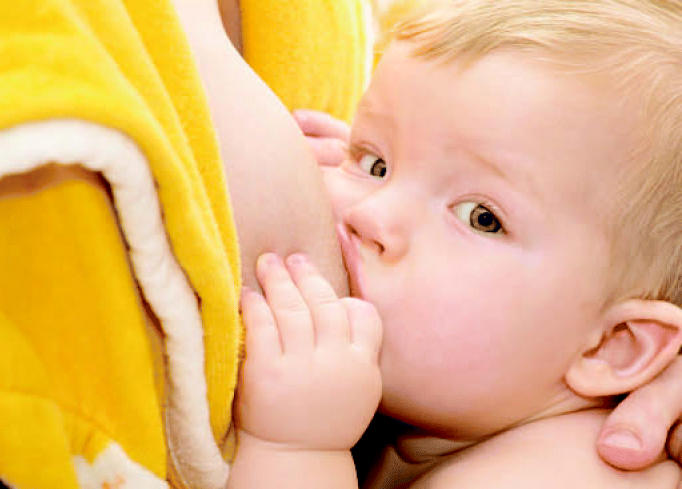
Lactation equation A new study shows that PFCs are transferred into breast milk at concentrations about 1% of maternal serum levels.

